# Peter Sykes MB, ChB, FRCPsych, DPM

**DOI:** 10.1192/bjb.2018.23

**Published:** 2018-08

**Authors:** Emma Savin

Formerly Consultant Psychiatrist, Greaves Hall Hospital, Banks, Southport, UK.


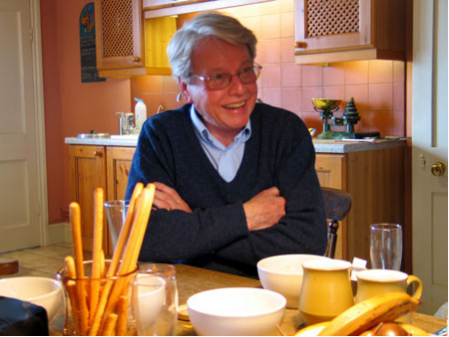


Peter Sykes, who died aged 86 in March 2017, was a pioneer in the field of the psychiatry of learning disorders. After postgraduate training, he held consultant posts in the north of England and in Scotland. He then spent 2 years in Canada from 1969 to 1971, where he was appointed Director of Mental Health Services for the Province of New Brunswick. He carried out a complete reorganisation of the mental health and mental subnormality services. The new service was based on community involvement and divisional responsibility. Throughout his working life, he championed a person-orientated approach to the care of patients with severe mental health problems and learning difficulties, moving away from the ‘asylum’ principle of care towards a more integrated, community-based approach that still recognised the important role of residential units.

On returning to England, he took up a post in Peterborough and Cambridge, where he again set up services in a general hospital unit rather than the then traditional model based on subnormality hospitals. On appointment to the Greaves Hall Hospital, Southport, in 1973, he provided a model for community-based services that was influential both locally and nationally. He was widely involved in the planning and design of new hospitals in the north of England and Scotland, always working in close association with the charity MIND.

Throughout his career, he carried out a considerable amount of forensic work. He also lectured widely, having, at various times, honorary lecturer appointments at the Universities of Liverpool and Aberdeen.

At the end of his clinical training in the Medical School of the University of Sheffield, he was the prize medallist in both clinical medicine and surgery. After psychiatric training, he was appointed to his first consultant post at the age of 29, being at that time the youngest doctor in England to be appointed as a consultant.

Peter saw medicine as an art rather than a science and had an open mind about new approaches to psychiatric care. Colleagues would often refer their complex patients to him, and he would use new techniques such as hypnotism to bring about dramatic improvements.

Always a practical man, as well as a plain-speaking Yorkshireman, Peter loved to find out how things worked and would spend hours tinkering with his old Morris Minor. His approach to these endeavours is best summed up by his motto ‘when all else fails, read the instructions’. As an example of his practical skill, while working in Scotland he devised a means of stopping bulls from falling over (a catastrophic event) and shared his technique with the local farmers.

He leaves his wife Jean, four daughters, seven grandchildren and five great-grandchildren.

